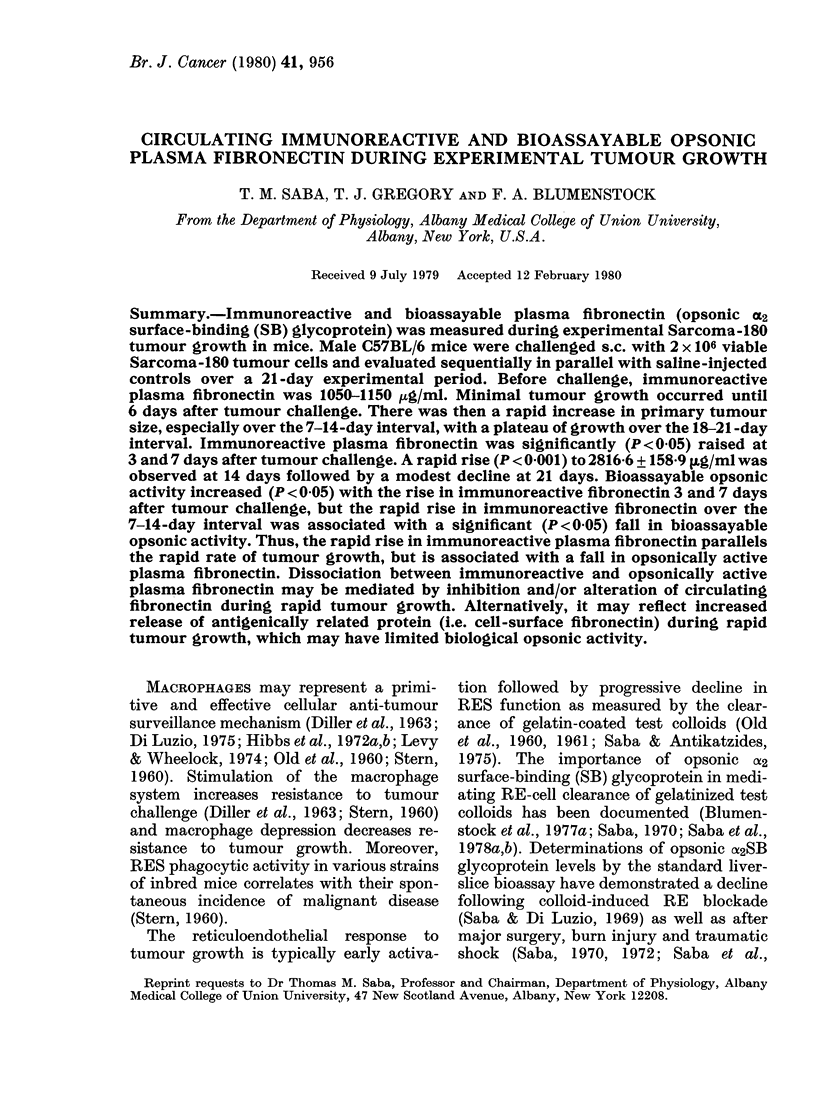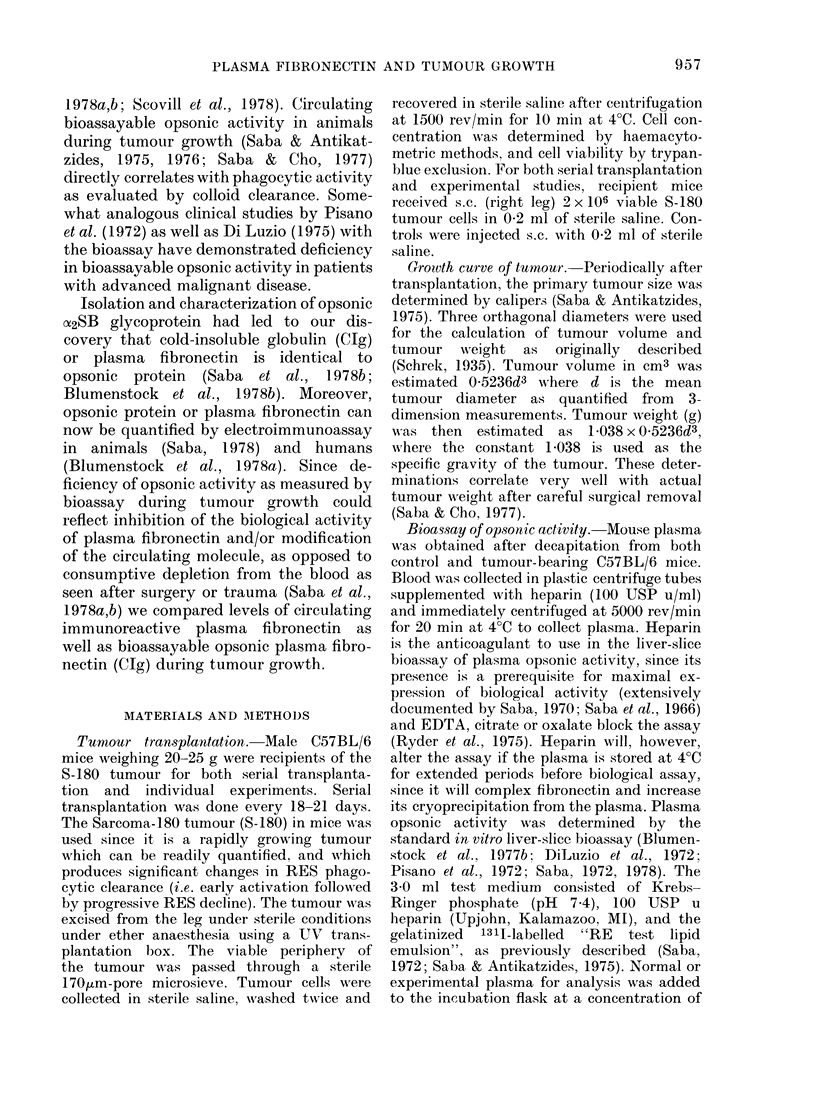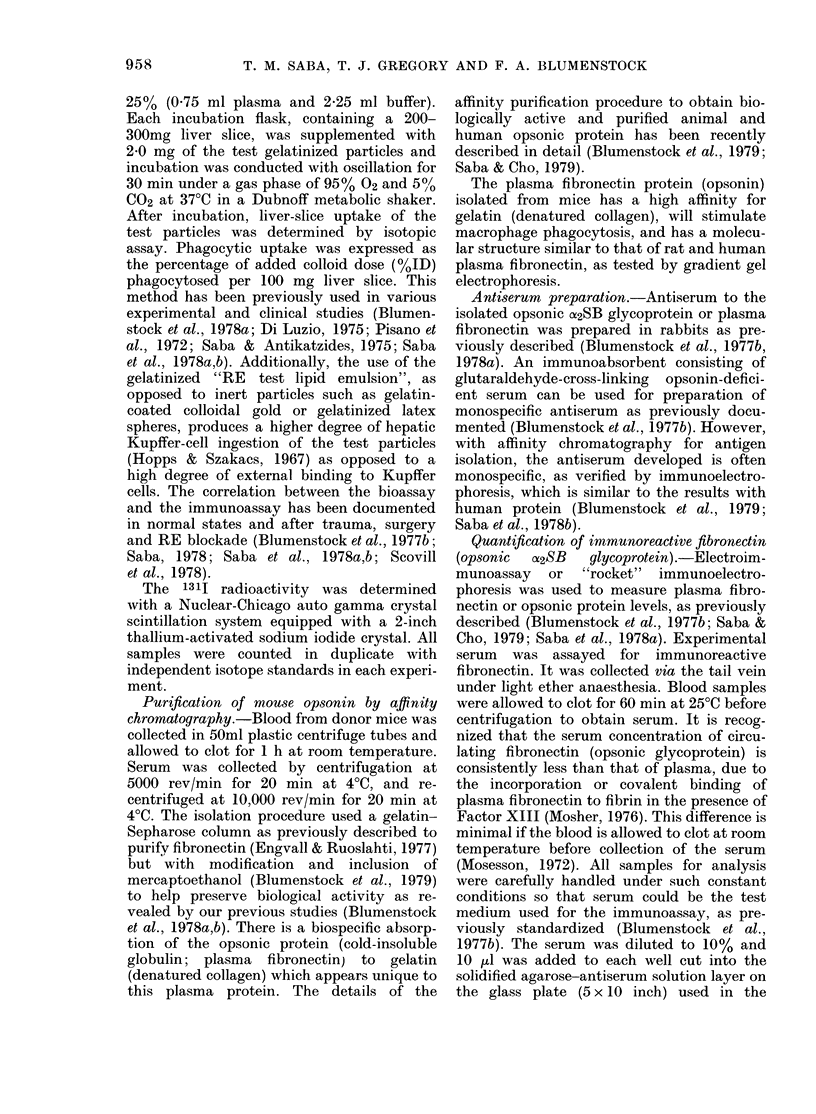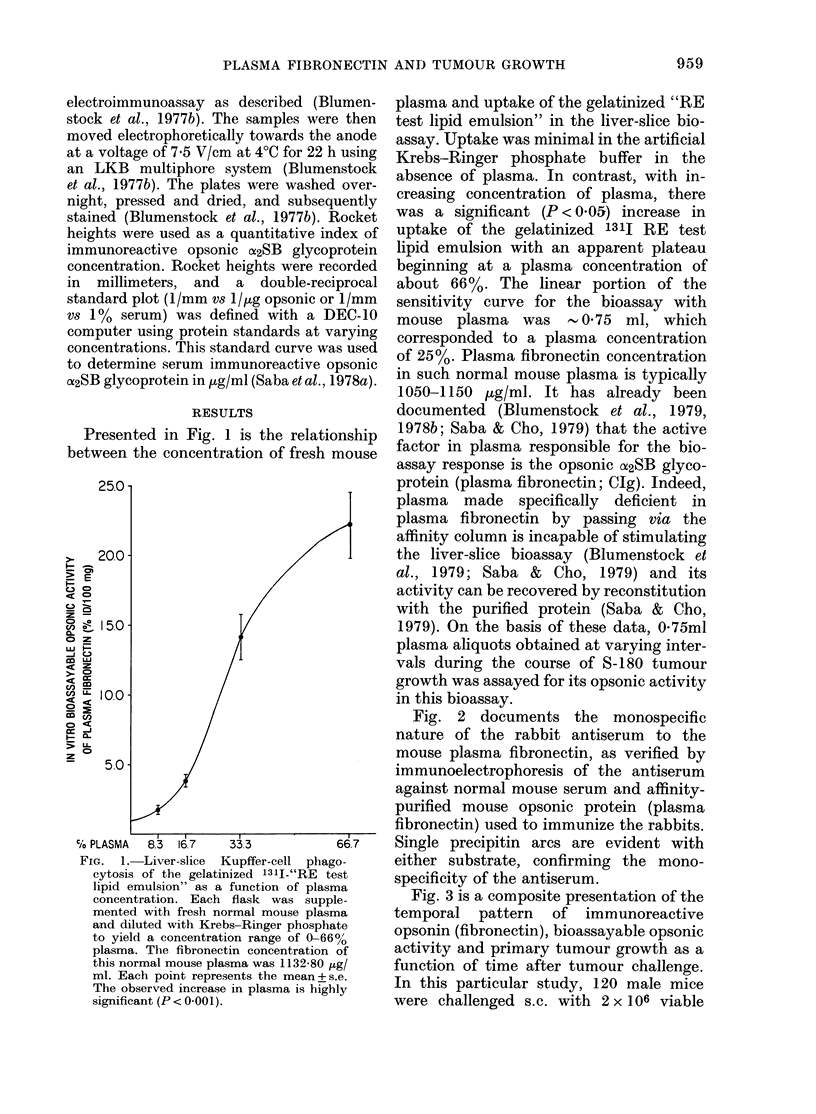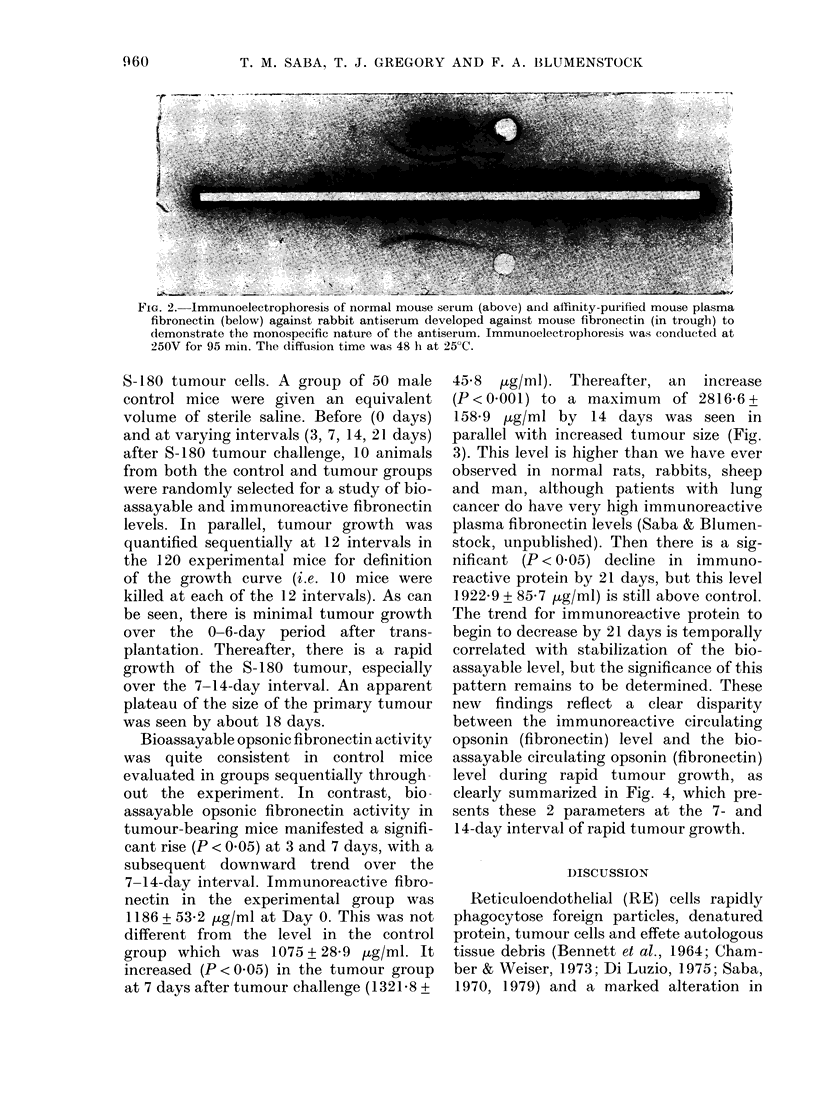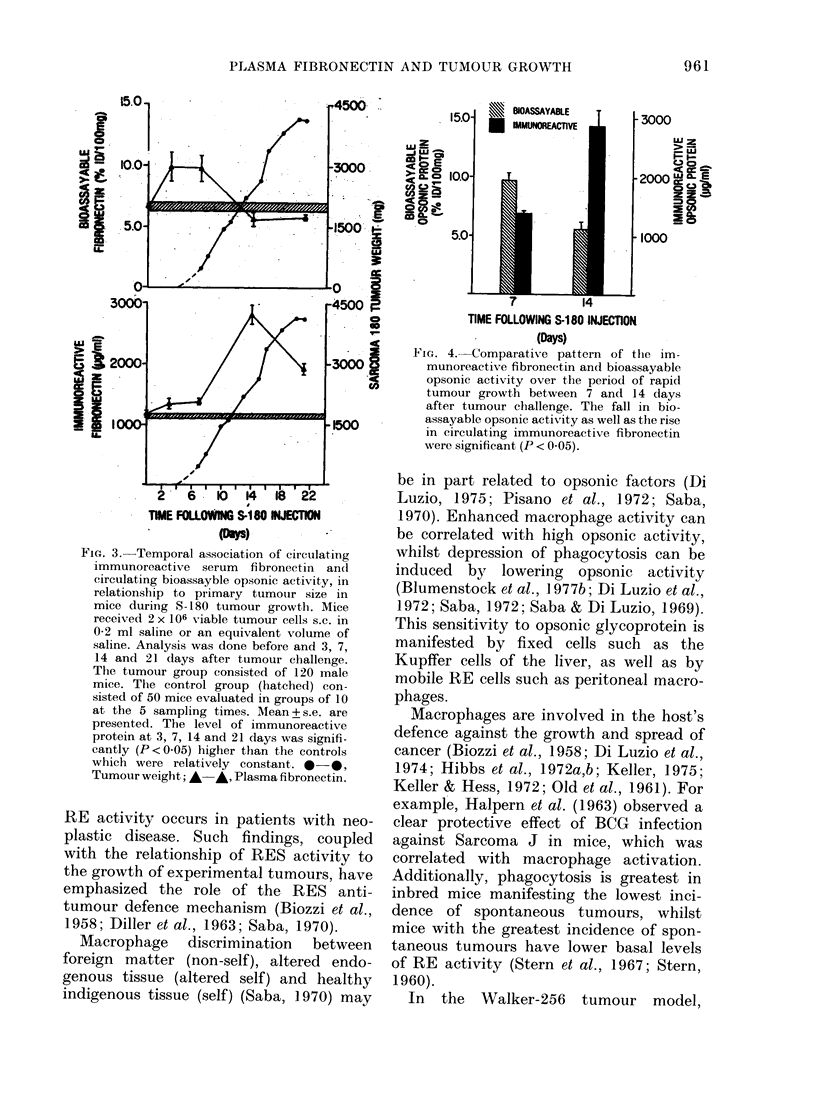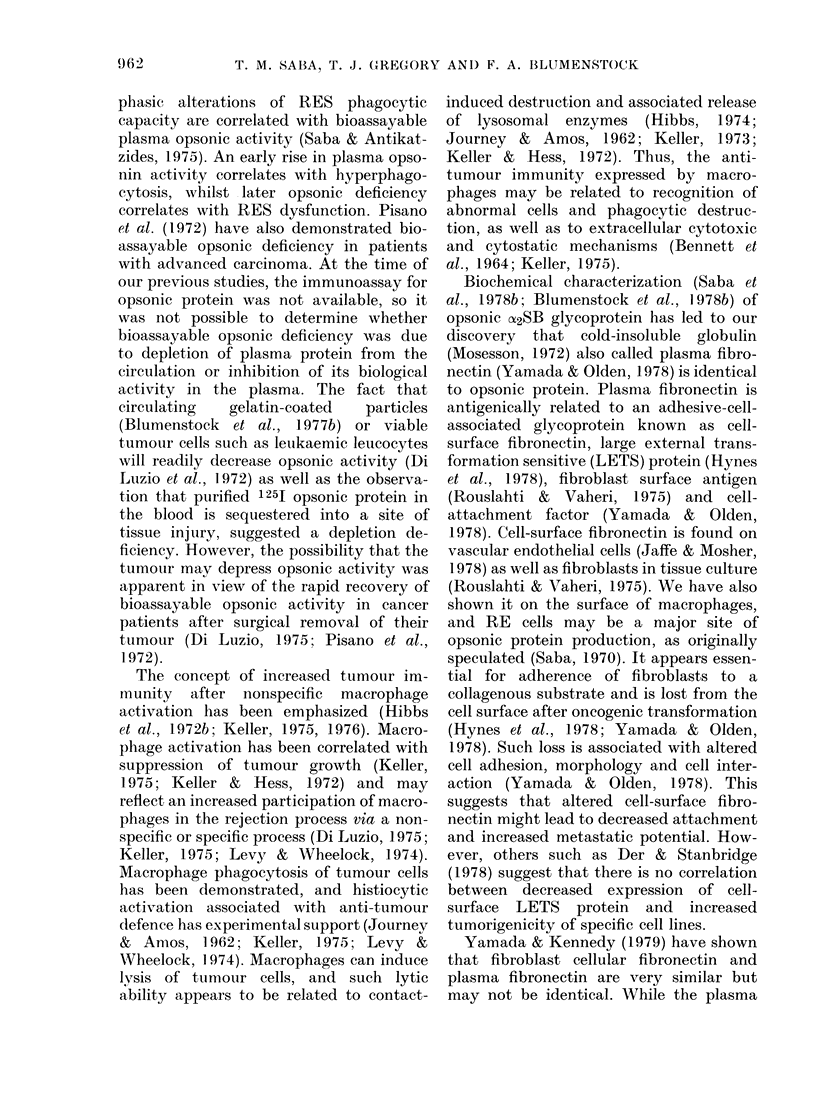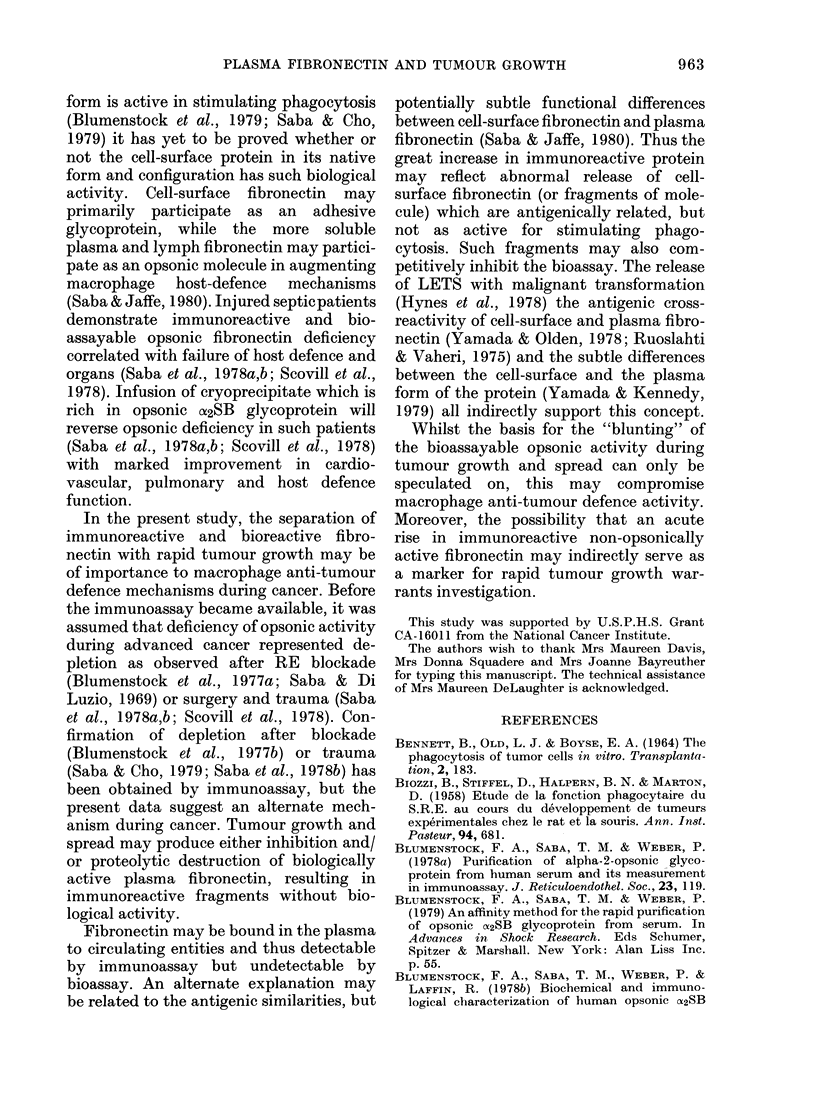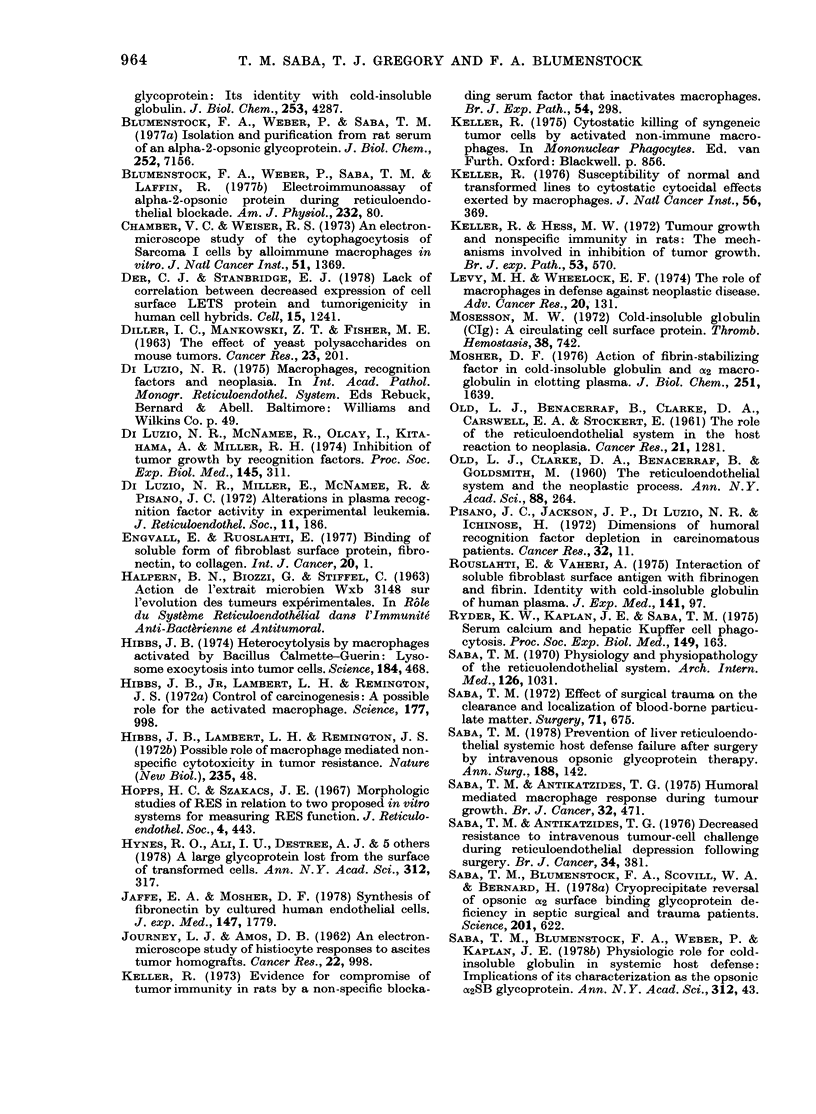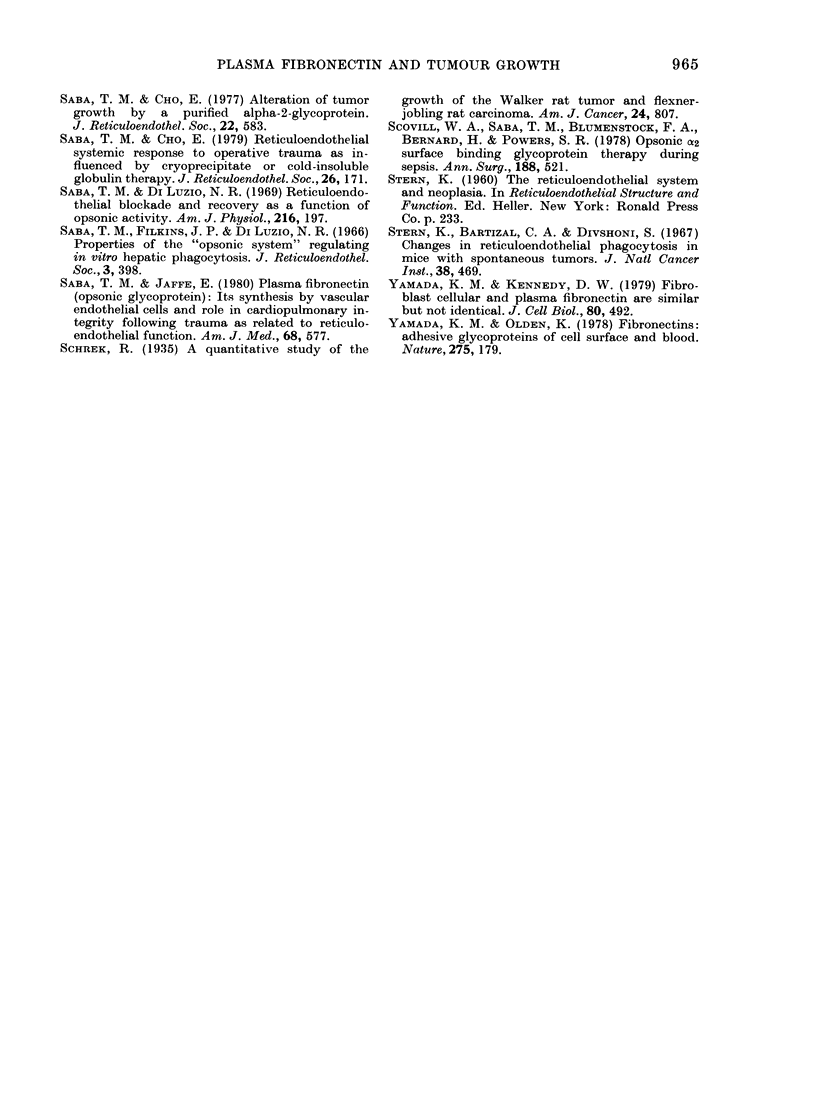# Circulating immunoreactive and bioassayable opsonic plasma fibronectin during experimental tumour growth

**DOI:** 10.1038/bjc.1980.174

**Published:** 1980-06

**Authors:** T. M. Saba, T. J. Gregory, F. A. Blumenstock

## Abstract

**Images:**


*Br. J. Cancer* (1980) **41**, 956

### CIRCULATING IMMUNOREACTIVE AND BIOASSAYABLE OPSONIC PLASMA FIBRONECTIN DURING EXPERIMENTAL TUMOUR GROWTH

T. M. SABA, T. J. GREGORY AND F. A. BLUMENSTOCK

*From the Department of Physiology, Albany Medical College of Union University, Albany, New York, U.S.A.*

Received 9 July 1979 Accepted 12 February 1980

**Summary.—Immunoreactive and bioassayable plasma fibronectin (opsonic $\alpha_2$ surface-binding (SB) glycoprotein) was measured during experimental Sarcoma-180 tumour growth in mice. Male C57BL/6 mice were challenged s.c. with $2 \times 10^6$ viable Sarcoma-180 tumour cells and evaluated sequentially in parallel with saline-injected controls over a 21-day experimental period. Before challenge, immunoreactive plasma fibronectin was 1050–1150 μg/ml. Minimal tumour growth occurred until 6 days after tumour challenge. There was then a rapid increase in primary tumour size, especially over the 7–14-day interval, with a plateau of growth over the 18–21-day interval. Immunoreactive plasma fibronectin was significantly ($P < 0{\cdot}05$) raised at 3 and 7 days after tumour challenge. A rapid rise ($P < 0{\cdot}001$) to 2816·6 ± 158·9 μg/ml was observed at 14 days followed by a modest decline at 21 days. Bioassayable opsonic activity increased ($P < 0{\cdot}05$) with the rise in immunoreactive fibronectin 3 and 7 days after tumour challenge, but the rapid rise in immunoreactive fibronectin over the 7–14-day interval was associated with a significant ($P < 0{\cdot}05$) fall in bioassayable opsonic activity. Thus, the rapid rise in immunoreactive plasma fibronectin parallels the rapid rate of tumour growth, but is associated with a fall in opsonically active plasma fibronectin. Dissociation between immunoreactive and opsonically active plasma fibronectin may be mediated by inhibition and/or alteration of circulating fibronectin during rapid tumour growth. Alternatively, it may reflect increased release of antigenically related protein (i.e. cell-surface fibronectin) during rapid tumour growth, which may have limited biological opsonic activity.**

MACROPHAGES may represent a primitive and effective cellular anti-tumour surveillance mechanism (Diller *et al.*, 1963; Di Luzio, 1975; Hibbs *et al.*, 1972*a,b*; Levy & Wheelock, 1974; Old *et al.*, 1960; Stern, 1960). Stimulation of the macrophage system increases resistance to tumour challenge (Diller *et al.*, 1963; Stern, 1960) and macrophage depression decreases resistance to tumour growth. Moreover, RES phagocytic activity in various strains of inbred mice correlates with their spontaneous incidence of malignant disease (Stern, 1960).

The reticuloendothelial response to tumour growth is typically early activation followed by progressive decline in RES function as measured by the clearance of gelatin-coated test colloids (Old *et al.*, 1960, 1961; Saba & Antikatzides, 1975). The importance of opsonic $\alpha_2$ surface-binding (SB) glycoprotein in mediating RE-cell clearance of gelatinized test colloids has been documented (Blumenstock *et al.*, 1977*a*; Saba, 1970; Saba *et al.*, 1978*a,b*). Determinations of opsonic $\alpha_2$SB glycoprotein levels by the standard liver-slice bioassay have demonstrated a decline following colloid-induced RE blockade (Saba & Di Luzio, 1969) as well as after major surgery, burn injury and traumatic shock (Saba, 1970, 1972; Saba *et al.*,

Reprint requests to Dr Thomas M. Saba, Professor and Chairman, Department of Physiology, Albany Medical College of Union University, 47 New Scotland Avenue, Albany, New York 12208.

1978*a*,*b*; Scovill *et al.*, 1978). Circulating bioassayable opsonic activity in animals during tumour growth (Saba & Antikatzides, 1975, 1976; Saba & Cho, 1977) directly correlates with phagocytic activity as evaluated by colloid clearance. Somewhat analogous clinical studies by Pisano *et al.* (1972) as well as Di Luzio (1975) with the bioassay have demonstrated deficiency in bioassayable opsonic activity in patients with advanced malignant disease.

Isolation and characterization of opsonic $\alpha_2$SB glycoprotein had led to our discovery that cold-insoluble globulin (CIg) or plasma fibronectin is identical to opsonic protein (Saba *et al.*, 1978*b*; Blumenstock *et al.*, 1978*b*). Moreover, opsonic protein or plasma fibronectin can now be quantified by electroimmunoassay in animals (Saba, 1978) and humans (Blumenstock *et al.*, 1978*a*). Since deficiency of opsonic activity as measured by bioassay during tumour growth could reflect inhibition of the biological activity of plasma fibronectin and/or modification of the circulating molecule, as opposed to consumptive depletion from the blood as seen after surgery or trauma (Saba *et al.*, 1978*a*,*b*) we compared levels of circulating immunoreactive plasma fibronectin as well as bioassayable opsonic plasma fibronectin (CIg) during tumour growth.

### MATERIALS AND METHODS

*Tumour transplantation.*—Male C57BL/6 mice weighing 20–25 g were recipients of the S-180 tumour for both serial transplantation and individual experiments. Serial transplantation was done every 18–21 days. The Sarcoma-180 tumour (S-180) in mice was used since it is a rapidly growing tumour which can be readily quantified, and which produces significant changes in RES phagocytic clearance (*i.e.* early activation followed by progressive RES decline). The tumour was excised from the leg under sterile conditions under ether anaesthesia using a UV transplantation box. The viable periphery of the tumour was passed through a sterile 170$\mu$m-pore microsieve. Tumour cells were collected in sterile saline, washed twice and recovered in sterile saline after centrifugation at 1500 rev/min for 10 min at 4°C. Cell concentration was determined by haemacytometric methods, and cell viability by trypan-blue exclusion. For both serial transplantation and experimental studies, recipient mice received s.c. (right leg) $2 \times 10^6$ viable S-180 tumour cells in 0·2 ml of sterile saline. Controls were injected s.c. with 0·2 ml of sterile saline.

*Growth curve of tumour.*—Periodically after transplantation, the primary tumour size was determined by calipers (Saba & Antikatzides, 1975). Three orthagonal diameters were used for the calculation of tumour volume and tumour weight as originally described (Schrek, 1935). Tumour volume in cm$^3$ was estimated $0{\cdot}5236d^3$ where $d$ is the mean tumour diameter as quantified from 3-dimension measurements. Tumour weight (g) was then estimated as $1{\cdot}038 \times 0{\cdot}5236d^3$, where the constant 1·038 is used as the specific gravity of the tumour. These determinations correlate very well with actual tumour weight after careful surgical removal (Saba & Cho, 1977).

*Bioassay of opsonic activity.*—Mouse plasma was obtained after decapitation from both control and tumour-bearing C57BL/6 mice. Blood was collected in plastic centrifuge tubes supplemented with heparin (100 USP u/ml) and immediately centrifuged at 5000 rev/min for 20 min at 4°C to collect plasma. Heparin is the anticoagulant to use in the liver-slice bioassay of plasma opsonic activity, since its presence is a prerequisite for maximal expression of biological activity (extensively documented by Saba, 1970; Saba *et al.*, 1966) and EDTA, citrate or oxalate block the assay (Ryder *et al.*, 1975). Heparin will, however, alter the assay if the plasma is stored at 4°C for extended periods before biological assay, since it will complex fibronectin and increase its cryoprecipitation from the plasma. Plasma opsonic activity was determined by the standard *in vitro* liver-slice bioassay (Blumenstock *et al.*, 1977*b*; DiLuzio *et al.*, 1972; Pisano *et al.*, 1972; Saba, 1972, 1978). The 3·0 ml test medium consisted of Krebs–Ringer phosphate (pH 7·4), 100 USP u heparin (Upjohn, Kalamazoo, MI), and the gelatinized $^{131}$I-labelled "RE test lipid emulsion", as previously described (Saba, 1972; Saba & Antikatzides, 1975). Normal or experimental plasma for analysis was added to the incubation flask at a concentration of

25% (0·75 ml plasma and 2·25 ml buffer). Each incubation flask, containing a 200–300mg liver slice, was supplemented with 2·0 mg of the test gelatinized particles and incubation was conducted with oscillation for 30 min under a gas phase of 95% $O_2$ and 5% $CO_2$ at 37°C in a Dubnoff metabolic shaker. After incubation, liver-slice uptake of the test particles was determined by isotopic assay. Phagocytic uptake was expressed as the percentage of added colloid dose (%ID) phagocytosed per 100 mg liver slice. This method has been previously used in various experimental and clinical studies (Blumenstock *et al.*, 1978*a*; Di Luzio, 1975; Pisano *et al.*, 1972; Saba & Antikatzides, 1975; Saba *et al.*, 1978*a*,*b*). Additionally, the use of the gelatinized "RE test lipid emulsion", as opposed to inert particles such as gelatin-coated colloidal gold or gelatinized latex spheres, produces a higher degree of hepatic Kupffer-cell ingestion of the test particles (Hopps & Szakacs, 1967) as opposed to a high degree of external binding to Kupffer cells. The correlation between the bioassay and the immunoassay has been documented in normal states and after trauma, surgery and RE blockade (Blumenstock *et al.*, 1977*b*; Saba, 1978; Saba *et al.*, 1978*a*,*b*; Scovill *et al.*, 1978).

The $^{131}I$ radioactivity was determined with a Nuclear-Chicago auto gamma crystal scintillation system equipped with a 2-inch thallium-activated sodium iodide crystal. All samples were counted in duplicate with independent isotope standards in each experiment.

*Purification of mouse opsonin by affinity chromatography.*—Blood from donor mice was collected in 50ml plastic centrifuge tubes and allowed to clot for 1 h at room temperature. Serum was collected by centrifugation at 5000 rev/min for 20 min at 4°C, and re-centrifuged at 10,000 rev/min for 20 min at 4°C. The isolation procedure used a gelatin–Sepharose column as previously described to purify fibronectin (Engvall & Ruoslahti, 1977) but with modification and inclusion of mercaptoethanol (Blumenstock *et al.*, 1979) to help preserve biological activity as revealed by our previous studies (Blumenstock *et al.*, 1978*a*,*b*). There is a biospecific absorption of the opsonic protein (cold-insoluble globulin; plasma fibronectin) to gelatin (denatured collagen) which appears unique to this plasma protein. The details of the affinity purification procedure to obtain biologically active and purified animal and human opsonic protein has been recently described in detail (Blumenstock *et al.*, 1979; Saba & Cho, 1979).

The plasma fibronectin protein (opsonin) isolated from mice has a high affinity for gelatin (denatured collagen), will stimulate macrophage phagocytosis, and has a molecular structure similar to that of rat and human plasma fibronectin, as tested by gradient gel electrophoresis.

*Antiserum preparation.*—Antiserum to the isolated opsonic $\alpha_2$SB glycoprotein or plasma fibronectin was prepared in rabbits as previously described (Blumenstock *et al.*, 1977*b*, 1978*a*). An immunoabsorbent consisting of glutaraldehyde-cross-linking opsonin-deficient serum can be used for preparation of monospecific antiserum as previously documented (Blumenstock *et al.*, 1977*b*). However, with affinity chromatography for antigen isolation, the antiserum developed is often monospecific, as verified by immunoelectrophoresis, which is similar to the results with human protein (Blumenstock *et al.*, 1979; Saba *et al.*, 1978*b*).

*Quantification of immunoreactive fibronectin (opsonic $\alpha_2$SB glycoprotein).*—Electroimmunoassay or "rocket" immunoelectrophoresis was used to measure plasma fibronectin or opsonic protein levels, as previously described (Blumenstock *et al.*, 1977*b*; Saba & Cho, 1979; Saba *et al.*, 1978*a*). Experimental serum was assayed for immunoreactive fibronectin. It was collected *via* the tail vein under light ether anaesthesia. Blood samples were allowed to clot for 60 min at 25°C before centrifugation to obtain serum. It is recognized that the serum concentration of circulating fibronectin (opsonic glycoprotein) is consistently less than that of plasma, due to the incorporation or covalent binding of plasma fibronectin to fibrin in the presence of Factor XIII (Mosher, 1976). This difference is minimal if the blood is allowed to clot at room temperature before collection of the serum (Mosesson, 1972). All samples for analysis were carefully handled under such constant conditions so that serum could be the test medium used for the immunoassay, as previously standardized (Blumenstock *et al.*, 1977*b*). The serum was diluted to 10% and 10 $\mu$l was added to each well cut into the solidified agarose–antiserum solution layer on the glass plate (5 × 10 inch) used in the electroimmunoassay as described (Blumenstock *et al.*, 1977*b*). The samples were then moved electrophoretically towards the anode at a voltage of 7·5 V/cm at 4°C for 22 h using an LKB multiphore system (Blumenstock *et al.*, 1977*b*). The plates were washed overnight, pressed and dried, and subsequently stained (Blumenstock *et al.*, 1977*b*). Rocket heights were used as a quantitative index of immunoreactive opsonic $\alpha_2$SB glycoprotein concentration. Rocket heights were recorded in millimeters, and a double-reciprocal standard plot (1/mm *vs* 1/$\mu$g opsonic or 1/mm *vs* 1% serum) was defined with a DEC-10 computer using protein standards at varying concentrations. This standard curve was used to determine serum immunoreactive opsonic $\alpha_2$SB glycoprotein in $\mu$g/ml (Saba *et al.*, 1978*a*).

### RESULTS

Presented in Fig. 1 is the relationship between the concentration of fresh mouse

FIG. 1.—Liver-slice Kupffer-cell phagocytosis of the gelatinized $^{131}$I-"RE test lipid emulsion" as a function of plasma concentration. Each flask was supplemented with fresh normal mouse plasma and diluted with Krebs–Ringer phosphate to yield a concentration range of 0–66% plasma. The fibronectin concentration of this normal mouse plasma was 1132·80 $\mu$g/ml. Each point represents the mean ± s.e. The observed increase in plasma is highly significant ($P < 0{\cdot}001$).

plasma and uptake of the gelatinized "RE test lipid emulsion" in the liver-slice bioassay. Uptake was minimal in the artificial Krebs–Ringer phosphate buffer in the absence of plasma. In contrast, with increasing concentration of plasma, there was a significant ($P < 0{\cdot}05$) increase in uptake of the gelatinized $^{131}$I RE test lipid emulsion with an apparent plateau beginning at a plasma concentration of about 66%. The linear portion of the sensitivity curve for the bioassay with mouse plasma was ~0·75 ml, which corresponded to a plasma concentration of 25%. Plasma fibronectin concentration in such normal mouse plasma is typically 1050–1150 $\mu$g/ml. It has already been documented (Blumenstock *et al.*, 1979, 1978*b*; Saba & Cho, 1979) that the active factor in plasma responsible for the bioassay response is the opsonic $\alpha_2$SB glycoprotein (plasma fibronectin; CIg). Indeed, plasma made specifically deficient in plasma fibronectin by passing *via* the affinity column is incapable of stimulating the liver-slice bioassay (Blumenstock *et al.*, 1979; Saba & Cho, 1979) and its activity can be recovered by reconstitution with the purified protein (Saba & Cho, 1979). On the basis of these data, 0·75ml plasma aliquots obtained at varying intervals during the course of S-180 tumour growth was assayed for its opsonic activity in this bioassay.

Fig. 2 documents the monospecific nature of the rabbit antiserum to the mouse plasma fibronectin, as verified by immunoelectrophoresis of the antiserum against normal mouse serum and affinity-purified mouse opsonic protein (plasma fibronectin) used to immunize the rabbits. Single precipitin arcs are evident with either substrate, confirming the monospecificity of the antiserum.

Fig. 3 is a composite presentation of the temporal pattern of immunoreactive opsonin (fibronectin), bioassayable opsonic activity and primary tumour growth as a function of time after tumour challenge. In this particular study, 120 male mice were challenged s.c. with $2 \times 10^6$ viable

FIG. 2.—Immunoelectrophoresis of normal mouse serum (above) and affinity-purified mouse plasma fibronectin (below) against rabbit antiserum developed against mouse fibronectin (in trough) to demonstrate the monospecific nature of the antiserum. Immunoelectrophoresis was conducted at 250V for 95 min. The diffusion time was 48 h at 25°C.

S-180 tumour cells. A group of 50 male control mice were given an equivalent volume of sterile saline. Before (0 days) and at varying intervals (3, 7, 14, 21 days) after S-180 tumour challenge, 10 animals from both the control and tumour groups were randomly selected for a study of bioassayable and immunoreactive fibronectin levels. In parallel, tumour growth was quantified sequentially at 12 intervals in the 120 experimental mice for definition of the growth curve (*i.e.* 10 mice were killed at each of the 12 intervals). As can be seen, there is minimal tumour growth over the 0–6-day period after transplantation. Thereafter, there is a rapid growth of the S-180 tumour, especially over the 7–14-day interval. An apparent plateau of the size of the primary tumour was seen by about 18 days.

Bioassayable opsonic fibronectin activity was quite consistent in control mice evaluated in groups sequentially throughout the experiment. In contrast, bioassayable opsonic fibronectin activity in tumour-bearing mice manifested a significant rise ($P < 0{\cdot}05$) at 3 and 7 days, with a subsequent downward trend over the 7–14-day interval. Immunoreactive fibronectin in the experimental group was $1186 \pm 53{\cdot}2$ $\mu$g/ml at Day 0. This was not different from the level in the control group which was $1075 \pm 28{\cdot}9$ $\mu$g/ml. It increased ($P < 0{\cdot}05$) in the tumour group at 7 days after tumour challenge ($1321{\cdot}8 \pm 45{\cdot}8$ $\mu$g/ml). Thereafter, an increase ($P < 0{\cdot}001$) to a maximum of $2816{\cdot}6 \pm 158{\cdot}9$ $\mu$g/ml by 14 days was seen in parallel with increased tumour size (Fig. 3). This level is higher than we have ever observed in normal rats, rabbits, sheep and man, although patients with lung cancer do have very high immunoreactive plasma fibronectin levels (Saba & Blumenstock, unpublished). Then there is a significant ($P < 0{\cdot}05$) decline in immunoreactive protein by 21 days, but this level $1922{\cdot}9 \pm 85{\cdot}7$ $\mu$g/ml) is still above control. The trend for immunoreactive protein to begin to decrease by 21 days is temporally correlated with stabilization of the bioassayable level, but the significance of this pattern remains to be determined. These new findings reflect a clear disparity between the immunoreactive circulating opsonin (fibronectin) level and the bioassayable circulating opsonin (fibronectin) level during rapid tumour growth, as clearly summarized in Fig. 4, which presents these 2 parameters at the 7- and 14-day interval of rapid tumour growth.

### DISCUSSION

Reticuloendothelial (RE) cells rapidly phagocytose foreign particles, denatured protein, tumour cells and effete autologous tissue debris (Bennett *et al.*, 1964; Chamber & Weiser, 1973; Di Luzio, 1975; Saba, 1970, 1979) and a marked alteration in

FIG. 3.—Temporal association of circulating immunoreactive serum fibronectin and circulating bioassayble opsonic activity, in relationship to primary tumour size in mice during S-180 tumour growth. Mice received $2 \times 10^6$ viable tumour cells s.c. in 0·2 ml saline or an equivalent volume of saline. Analysis was done before and 3, 7, 14 and 21 days after tumour challenge. The tumour group consisted of 120 male mice. The control group (hatched) consisted of 50 mice evaluated in groups of 10 at the 5 sampling times. Mean ± s.e. are presented. The level of immunoreactive protein at 3, 7, 14 and 21 days was significantly ($P < 0·05$) higher than the controls which were relatively constant. ●—●, Tumour weight; ▲—▲, Plasma fibronectin.

FIG. 4.—Comparative pattern of the immunoreactive fibronectin and bioassayable opsonic activity over the period of rapid tumour growth between 7 and 14 days after tumour challenge. The fall in bioassayable opsonic activity as well as the rise in circulating immunoreactive fibronectin were significant ($P < 0·05$).

RE activity occurs in patients with neoplastic disease. Such findings, coupled with the relationship of RES activity to the growth of experimental tumours, have emphasized the role of the RES antitumour defence mechanism (Biozzi *et al.*, 1958; Diller *et al.*, 1963; Saba, 1970).

Macrophage discrimination between foreign matter (non-self), altered endogenous tissue (altered self) and healthy indigenous tissue (self) (Saba, 1970) may be in part related to opsonic factors (Di Luzio, 1975; Pisano *et al.*, 1972; Saba, 1970). Enhanced macrophage activity can be correlated with high opsonic activity, whilst depression of phagocytosis can be induced by lowering opsonic activity (Blumenstock *et al.*, 1977*b*; Di Luzio *et al.*, 1972; Saba, 1972; Saba & Di Luzio, 1969). This sensitivity to opsonic glycoprotein is manifested by fixed cells such as the Kupffer cells of the liver, as well as by mobile RE cells such as peritoneal macrophages.

Macrophages are involved in the host's defence against the growth and spread of cancer (Biozzi *et al.*, 1958; Di Luzio *et al.*, 1974; Hibbs *et al.*, 1972*a,b*; Keller, 1975; Keller & Hess, 1972; Old *et al.*, 1961). For example, Halpern *et al.* (1963) observed a clear protective effect of BCG infection against Sarcoma J in mice, which was correlated with macrophage activation. Additionally, phagocytosis is greatest in inbred mice manifesting the lowest incidence of spontaneous tumours, whilst mice with the greatest incidence of spontaneous tumours have lower basal levels of RE activity (Stern *et al.*, 1967; Stern, 1960).

In the Walker-256 tumour model, phasic alterations of RES phagocytic capacity are correlated with bioassayable plasma opsonic activity (Saba & Antikatzides, 1975). An early rise in plasma opsonin activity correlates with hyperphagocytosis, whilst later opsonic deficiency correlates with RES dysfunction. Pisano *et al.* (1972) have also demonstrated bioassayable opsonic deficiency in patients with advanced carcinoma. At the time of our previous studies, the immunoassay for opsonic protein was not available, so it was not possible to determine whether bioassayable opsonic deficiency was due to depletion of plasma protein from the circulation or inhibition of its biological activity in the plasma. The fact that circulating gelatin-coated particles (Blumenstock *et al.*, 1977*b*) or viable tumour cells such as leukaemic leucocytes will readily decrease opsonic activity (Di Luzio *et al.*, 1972) as well as the observation that purified $^{125}I$ opsonic protein in the blood is sequestered into a site of tissue injury, suggested a depletion deficiency. However, the possibility that the tumour may depress opsonic activity was apparent in view of the rapid recovery of bioassayable opsonic activity in cancer patients after surgical removal of their tumour (Di Luzio, 1975; Pisano *et al.*, 1972).

The concept of increased tumour immunity after nonspecific macrophage activation has been emphasized (Hibbs *et al.*, 1972*b*; Keller, 1975, 1976). Macrophage activation has been correlated with suppression of tumour growth (Keller, 1975; Keller & Hess, 1972) and may reflect an increased participation of macrophages in the rejection process *via* a nonspecific or specific process (Di Luzio, 1975; Keller, 1975; Levy & Wheelock, 1974). Macrophage phagocytosis of tumour cells has been demonstrated, and histiocytic activation associated with anti-tumour defence has experimental support (Journey & Amos, 1962; Keller, 1975; Levy & Wheelock, 1974). Macrophages can induce lysis of tumour cells, and such lytic ability appears to be related to contact-induced destruction and associated release of lysosomal enzymes (Hibbs, 1974; Journey & Amos, 1962; Keller, 1973; Keller & Hess, 1972). Thus, the anti-tumour immunity expressed by macrophages may be related to recognition of abnormal cells and phagocytic destruction, as well as to extracellular cytotoxic and cytostatic mechanisms (Bennett *et al.*, 1964; Keller, 1975).

Biochemical characterization (Saba *et al.*, 1978*b*; Blumenstock *et al.*, 1978*b*) of opsonic $\alpha_2$SB glycoprotein has led to our discovery that cold-insoluble globulin (Mosesson, 1972) also called plasma fibronectin (Yamada & Olden, 1978) is identical to opsonic protein. Plasma fibronectin is antigenically related to an adhesive-cell-associated glycoprotein known as cell-surface fibronectin, large external transformation sensitive (LETS) protein (Hynes *et al.*, 1978), fibroblast surface antigen (Rouslahti & Vaheri, 1975) and cell-attachment factor (Yamada & Olden, 1978). Cell-surface fibronectin is found on vascular endothelial cells (Jaffe & Mosher, 1978) as well as fibroblasts in tissue culture (Rouslahti & Vaheri, 1975). We have also shown it on the surface of macrophages, and RE cells may be a major site of opsonic protein production, as originally speculated (Saba, 1970). It appears essential for adherence of fibroblasts to a collagenous substrate and is lost from the cell surface after oncogenic transformation (Hynes *et al.*, 1978; Yamada & Olden, 1978). Such loss is associated with altered cell adhesion, morphology and cell interaction (Yamada & Olden, 1978). This suggests that altered cell-surface fibronectin might lead to decreased attachment and increased metastatic potential. However, others such as Der & Stanbridge (1978) suggest that there is no correlation between decreased expression of cell-surface LETS protein and increased tumorigenicity of specific cell lines.

Yamada & Kennedy (1979) have shown that fibroblast cellular fibronectin and plasma fibronectin are very similar but may not be identical. While the plasma form is active in stimulating phagocytosis (Blumenstock *et al.*, 1979; Saba & Cho, 1979) it has yet to be proved whether or not the cell-surface protein in its native form and configuration has such biological activity. Cell-surface fibronectin may primarily participate as an adhesive glycoprotein, while the more soluble plasma and lymph fibronectin may participate as an opsonic molecule in augmenting macrophage host-defence mechanisms (Saba & Jaffe, 1980). Injured septic patients demonstrate immunoreactive and bioassayable opsonic fibronectin deficiency correlated with failure of host defence and organs (Saba *et al.*, 1978*a,b*; Scovill *et al.*, 1978). Infusion of cryoprecipitate which is rich in opsonic $\alpha_2$SB glycoprotein will reverse opsonic deficiency in such patients (Saba *et al.*, 1978*a,b*; Scovill *et al.*, 1978) with marked improvement in cardiovascular, pulmonary and host defence function.

In the present study, the separation of immunoreactive and bioreactive fibronectin with rapid tumour growth may be of importance to macrophage anti-tumour defence mechanisms during cancer. Before the immunoassay became available, it was assumed that deficiency of opsonic activity during advanced cancer represented depletion as observed after RE blockade (Blumenstock *et al.*, 1977*a*; Saba & Di Luzio, 1969) or surgery and trauma (Saba *et al.*, 1978*a,b*; Scovill *et al.*, 1978). Confirmation of depletion after blockade (Blumenstock *et al.*, 1977*b*) or trauma (Saba & Cho, 1979; Saba *et al.*, 1978*b*) has been obtained by immunoassay, but the present data suggest an alternate mechanism during cancer. Tumour growth and spread may produce either inhibition and/or proteolytic destruction of biologically active plasma fibronectin, resulting in immunoreactive fragments without biological activity.

Fibronectin may be bound in the plasma to circulating entities and thus detectable by immunoassay but undetectable by bioassay. An alternate explanation may be related to the antigenic similarities, but potentially subtle functional differences between cell-surface fibronectin and plasma fibronectin (Saba & Jaffe, 1980). Thus the great increase in immunoreactive protein may reflect abnormal release of cell-surface fibronectin (or fragments of molecule) which are antigenically related, but not as active for stimulating phagocytosis. Such fragments may also competitively inhibit the bioassay. The release of LETS with malignant transformation (Hynes *et al.*, 1978) the antigenic cross-reactivity of cell-surface and plasma fibronectin (Yamada & Olden, 1978; Ruoslahti & Vaheri, 1975) and the subtle differences between the cell-surface and the plasma form of the protein (Yamada & Kennedy, 1979) all indirectly support this concept.

Whilst the basis for the "blunting" of the bioassayable opsonic activity during tumour growth and spread can only be speculated on, this may compromise macrophage anti-tumour defence activity. Moreover, the possibility that an acute rise in immunoreactive non-opsonically active fibronectin may indirectly serve as a marker for rapid tumour growth warrants investigation.

This study was supported by U.S.P.H.S. Grant CA-16011 from the National Cancer Institute.

The authors wish to thank Mrs Maureen Davis, Mrs Donna Squadere and Mrs Joanne Bayreuther for typing this manuscript. The technical assistance of Mrs Maureen DeLaughter is acknowledged.

### REFERENCES

BENNETT, B., OLD, L. J. & BOYSE, E. A. (1964) The phagocytosis of tumor cells *in vitro*. *Transplantation*, **2**, 183.

BIOZZI, B., STIFFEL, D., HALPERN, B. N. & MARTON, D. (1958) Etude de la fonction phagocytaire du S.R.E. au cours du développement de tumeurs expérimentales chez le rat et la souris. *Ann. Inst. Pasteur*, **94**, 681.

BLUMENSTOCK, F. A., SABA, T. M. & WEBER, P. (1978*a*) Purification of alpha-2-opsonic glycoprotein from human serum and its measurement in immunoassay. *J. Reticuloendothel. Soc.*, **23**, 119.

BLUMENSTOCK, F. A., SABA, T. M. & WEBER, P. (1979) An affinity method for the rapid purification of opsonic $\alpha_2$SB glycoprotein from serum. In *Advances in Shock Research*. Eds Schumer, Spitzer & Marshall. New York: Alan Liss Inc. p. 55.

BLUMENSTOCK, F. A., SABA, T. M., WEBER, P. & LAFFIN, R. (1978*b*) Biochemical and immunological characterization of human opsonic $\alpha_2$SB

glycoprotein: Its identity with cold-insoluble globulin. *J. Biol. Chem.*, **253**, 4287.

BLUMENSTOCK, F. A., WEBER, P. & SABA, T. M. (1977*a*) Isolation and purification from rat serum of an alpha-2-opsonic glycoprotein. *J. Biol. Chem.*, **252**, 7156.

BLUMENSTOCK, F. A., WEBER, P., SABA, T. M. & LAFFIN, R. (1977*b*) Electroimmunoassay of alpha-2-opsonic protein during reticuloendothelial blockade. *Am. J. Physiol.*, **232**, 80.

CHAMBER, V. C. & WEISER, R. S. (1973) An electron-microscope study of the cytophagocytosis of Sarcoma I cells by alloimmune macrophages *in vitro*. *J. Natl Cancer Inst.*, **51**, 1369.

DER, C. J. & STANBRIDGE, E. J. (1978) Lack of correlation between decreased expression of cell surface LETS protein and tumorigenicity in human cell hybrids. *Cell*, **15**, 1241.

DILLER, I. C., MANKOWSKI, Z. T. & FISHER, M. E. (1963) The effect of yeast polysaccharides on mouse tumors. *Cancer Res.*, **23**, 201.

DI LUZIO, N. R. (1975) Macrophages, recognition factors and neoplasia. In *Int. Acad. Pathol. Monogr. Reticuloendothel. System*. Eds Rebuck, Bernard & Abell. Baltimore: Williams and Wilkins Co. p. 49.

DI LUZIO, N. R., MCNAMEE, R., OLCAY, I., KITAHAMA, A. & MILLER, R. H. (1974) Inhibition of tumor growth by recognition factors. *Proc. Soc. Exp. Biol. Med.*, **145**, 311.

DI LUZIO, N. R., MILLER, E., MCNAMEE, R. & PISANO, J. C. (1972) Alterations in plasma recognition factor activity in experimental leukemia. *J. Reticuloendothel. Soc.*, **11**, 186.

ENGVALL, E. & RUOSLAHTI, E. (1977) Binding of soluble form of fibroblast surface protein, fibronectin, to collagen. *Int. J. Cancer*, **20**, 1.

HALPERN, B. N., BIOZZI, G. & STIFFEL, C. (1963) Action de l'extrait microbien Wxb 3148 sur l'evolution des tumeurs expèrimentales. In *Rôle du Système Reticuloendothèlial dans l'Immunité Anti-Bactèrienne et Antitumoral*.

HIBBS, J. B. (1974) Heterocytolysis by macrophages activated by Bacillus Calmette–Guerin: Lysosome exocytosis into tumor cells. *Science*, **184**, 468.

HIBBS, J. B., JR, LAMBERT, L. H. & REMINGTON, J. S. (1972*a*) Control of carcinogenesis: A possible role for the activated macrophage. *Science*, **177**, 998.

HIBBS, J. B., LAMBERT, L. H. & REMINGTON, J. S. (1972*b*) Possible role of macrophage mediated nonspecific cytotoxicity in tumor resistance. *Nature (New Biol.)*, **235**, 48.

HOPPS, H. C. & SZAKACS, J. E. (1967) Morphologic studies of RES in relation to two proposed *in vitro* systems for measuring RES function. *J. Reticuloendothel. Soc.*, **4**, 443.

HYNES, R. O., ALI, I. U., DESTREE, A. J. & 5 others (1978) A large glycoprotein lost from the surface of transformed cells. *Ann. N.Y. Acad. Sci.*, **312**, 317.

JAFFE, E. A. & MOSHER, D. F. (1978) Synthesis of fibronectin by cultured human endothelial cells. *J. exp. Med.*, **147**, 1779.

JOURNEY, L. J. & AMOS, D. B. (1962) An electron-microscope study of histiocyte responses to ascites tumor homografts. *Cancer Res.*, **22**, 998.

KELLER, R. (1973) Evidence for compromise of tumor immunity in rats by a non-specific blocking serum factor that inactivates macrophages. *Br. J. Exp. Path.*, **54**, 298.

KELLER, R. (1975) Cytostatic killing of syngeneic tumor cells by activated non-immune macrophages. In *Mononuclear Phagocytes*. Ed. van Furth. Oxford: Blackwell. p. 856.

KELLER, R. (1976) Susceptibility of normal and transformed lines to cytostatic cytocidal effects exerted by macrophages. *J. Natl Cancer Inst.*, **56**, 369.

KELLER, R. & HESS, M. W. (1972) Tumour growth and nonspecific immunity in rats: The mechanisms involved in inhibition of tumor growth. *Br. J. exp. Path.*, **53**, 570.

LEVY, M. H. & WHEELOCK, E. F. (1974) The role of macrophages in defense against neoplastic disease. *Adv. Cancer Res.*, **20**, 131.

MOSESSON, M. W. (1972) Cold-insoluble globulin (CIg): A circulating cell surface protein. *Thromb. Hemostasis*, **38**, 742.

MOSHER, D. F. (1976) Action of fibrin-stabilizing factor in cold-insoluble globulin and $\alpha_2$ macroglobulin in clotting plasma. *J. Biol. Chem.*, **251**, 1639.

OLD, L. J., BENACERRAF, B., CLARKE, D. A., CARSWELL, E. A. & STOCKERT, E. (1961) The role of the reticuloendothelial system in the host reaction to neoplasia. *Cancer Res.*, **21**, 1281.

OLD, L. J., CLARKE, D. A., BENACERRAF, B. & GOLDSMITH, M. (1960) The reticuloendothelial system and the neoplastic process. *Ann. N.Y. Acad. Sci.*, **88**, 264.

PISANO, J. C., JACKSON, J. P., DI LUZIO, N. R. & ICHINOSE, H. (1972) Dimensions of humoral recognition factor depletion in carcinomatous patients. *Cancer Res.*, **32**, 11.

ROUSLAHTI, E. & VAHERI, A. (1975) Interaction of soluble fibroblast surface antigen with fibrinogen and fibrin. Identity with cold-insoluble globulin of human plasma. *J. Exp. Med.*, **141**, 97.

RYDER, K. W., KAPLAN, J. E. & SABA, T. M. (1975) Serum calcium and hepatic Kupffer cell phagocytosis. *Proc. Soc. Exp. Biol. Med.*, **149**, 163.

SABA, T. M. (1970) Physiology and physiopathology of the reticuolendothelial system. *Arch. Intern. Med.*, **126**, 1031.

SABA, T. M. (1972) Effect of surgical trauma on the clearance and localization of blood-borne particulate matter. *Surgery*, **71**, 675.

SABA, T. M. (1978) Prevention of liver reticuloendothelial systemic host defense failure after surgery by intravenous opsonic glycoprotein therapy. *Ann. Surg.*, **188**, 142.

SABA, T. M. & ANTIKATZIDES, T. G. (1975) Humoral mediated macrophage response during tumour growth. *Br. J. Cancer*, **32**, 471.

SABA, T. M. & ANTIKATZIDES, T. G. (1976) Decreased resistance to intravenous tumour-cell challenge during reticuloendothelial depression following surgery. *Br. J. Cancer*, **34**, 381.

SABA, T. M., BLUMENSTOCK, F. A., SCOVILL, W. A. & BERNARD, H. (1978*a*) Cryoprecipitate reversal of opsonic $\alpha_2$ surface binding glycoprotein deficiency in septic surgical and trauma patients. *Science*, **201**, 622.

SABA, T. M., BLUMENSTOCK, F. A., WEBER, P. & KAPLAN, J. E. (1978*b*) Physiologic role for cold-insoluble globulin in systemic host defense: Implications of its characterization as the opsonic $\alpha_2$SB glycoprotein. *Ann. N.Y. Acad. Sci.*, **312**, 43.

SABA, T. M. & CHO, E. (1977) Alteration of tumor growth by a purified alpha-2-glycoprotein. *J. Reticuloendothel. Soc.*, **22**, 583.

SABA, T. M. & CHO, E. (1979) Reticuloendothelial systemic response to operative trauma as influenced by cryoprecipitate or cold-insoluble globulin therapy. *J. Reticuloendothel. Soc.*, **26**, 171.

SABA, T. M. & DI LUZIO, N. R. (1969) Reticuloendothelial blockade and recovery as a function of opsonic activity. *Am. J. Physiol.*, **216**, 197.

SABA, T. M., FILKINS, J. P. & DI LUZIO, N. R. (1966) Properties of the "opsonic system" regulating *in vitro* hepatic phagocytosis. *J. Reticuloendothel. Soc.*, **3**, 398.

SABA, T. M. & JAFFE, E. (1980) Plasma fibronectin (opsonic glycoprotein): Its synthesis by vascular endothelial cells and role in cardiopulmonary integrity following trauma as related to reticuloendothelial function. *Am. J. Med.*, **68**, 577.

SCHREK, R. (1935) A quantitative study of the growth of the Walker rat tumor and flexner-jobling rat carcinoma. *Am. J. Cancer*, **24**, 807.

SCOVILL, W. A., SABA, T. M., BLUMENSTOCK, F. A., BERNARD, H. & POWERS, S. R. (1978) Opsonic $\alpha_2$ surface binding glycoprotein therapy during sepsis. *Ann. Surg.*, **188**, 521.

STERN, K. (1960) The reticuloendothelial system and neoplasia. In *Reticuloendothelial Structure and Function*. Ed. Heller. New York: Ronald Press Co. p. 233.

STERN, K., BARTIZAL, C. A. & DIVSHONI, S. (1967) Changes in reticuloendothelial phagocytosis in mice with spontaneous tumors. *J. Natl Cancer Inst.*, **38**, 469.

YAMADA, K. M. & KENNEDY, D. W. (1979) Fibroblast cellular and plasma fibronectin are similar but not identical. *J. Cell Biol.*, **80**, 492.

YAMADA, K. M. & OLDEN, K. (1978) Fibronectins: adhesive glycoproteins of cell surface and blood. *Nature*, **275**, 179.